# Supplementary material for: High Efficiency of Low Dose Preparations of an Inactivated Lumpy Skin Disease Virus Vaccine Candidate
Source: Vaccines (Basel). 2022 Jun 27;10(7):1029. doi: 10.3390/vaccines10071029 (PMC9319008; doi:10.3390/vaccines10071029)
Supplement: Supplementary file 1 [file vaccines-10-01029-s001.zip › vaccines-1663097-supplementary.pdf]

Table S1. Viral genome load (Cq values) of organ samples taken during necropsy.

Cattle		Cervical lymph node	Mediastinal lymph node	Liver	Lung
Group A: undiluted antigen	R-701	no Cq	no Cq	no Cq	no Cq
	R-717	no Cq	no Cq	no Cq	no Cq
	R-650	no Cq	no Cq	no Cq	no Cq
	R-725	no Cq	no Cq	no Cq	no Cq
	R-765	no Cq	no Cq	no Cq	no Cq
	R-756	no Cq	no Cq	no Cq	no Cq
Group B: antigen diluted 1:10	R-722	no Cq	no Cq	no Cq	no Cq
	R-689	no Cq	no Cq	no Cq	no Cq
	R-729	no Cq	no Cq	no Cq	no Cq
	R-759	no Cq	no Cq	no Cq	no Cq
	R-987	no Cq	no Cq	no Cq	no Cq
	R-993	no Cq	no Cq	no Cq	no Cq
Group C: antigen diluted 1:100	R-672	no Cq	no Cq	n.t.	no Cq
	R-643	no Cq	no Cq	no Cq	no Cq
	R-703	no Cq	no Cq	no Cq	no Cq
	R-727	no Cq	no Cq	no Cq	no Cq
	R-760	no Cq	no Cq	no Cq	no Cq
	R-994	no Cq	no Cq	no Cq	no Cq
Group D: challenge control	R-728	27.0	32.2	no Cq	31.1
	R-714	no Cq	no Cq	no Cq	no Cq
	R-684	no Cq	no Cq	no Cq	no Cq
	R-762	27.3	34.7	no Cq	24.8
	R-988	29.4	37.3	no Cq	32.3

n.t. indicates sample not taken.
